# Soluble Markers of Antibody Secreting Cell Function as Predictors of Infection Risk in Rheumatoid Arthritis

**DOI:** 10.1155/2019/3658215

**Published:** 2019-04-28

**Authors:** Maria J. Gutierrez, Stephen V. Desiderio, Nae-Yuh Wang, Erika Darrah, Laura Cappelli, Gustavo Nino, Michelle Jones, Clifton O. Bingham

**Affiliations:** ^1^Division of Pediatric Allergy and Immunology, Johns Hopkins University School of Medicine, Baltimore, MD, USA; ^2^Department of Molecular Biology and Genetics, Johns Hopkins University School of Medicine, Baltimore, MD, USA; ^3^Department of Medicine, Division of General Internal Medicine, Johns Hopkins University School of Medicine, Baltimore, MD, USA; ^4^Department of Biostatistics, Johns Hopkins Bloomberg School of Public Health, Baltimore, MD, USA; ^5^Department of Epidemiology, Johns Hopkins Bloomberg School of Public Health, Baltimore, MD, USA; ^6^Division of Rheumatology, Johns Hopkins University School of Medicine, Baltimore, MD, USA; ^7^Division of Pulmonary and Sleep Medicine, Children's National Medical Center, Washington, DC, USA; ^8^Division of Allergy and Clinical Immunology, Johns Hopkins University School of Medicine, Baltimore, MD, USA

## Abstract

**Background:**

Rheumatoid arthritis (RA) is a systemic autoimmune disease associated with immune dysregulation and increased risk of infections. The presence of autoantibodies and immunoglobulin abnormalities indicates B-cell and antibody-secreting cell (ASC) dysfunction. We hypothesize that soluble factors associated with B-cell and ASC activity are decreased in RA patients and that this is linked to higher susceptibility to infections.

**Methods:**

Using the Johns Hopkins Arthritis Cohort and Biorepository, we contrasted serum protein levels of soluble factors involved in B-cell activation (CD40, CD40L) and B-cell/ASC homing (CXCL10, CXCL11, and CXCL13) or survival (BAFF, APRIL, TACI, and BCMA) in 10 healthy subjects and 23 adult RA patients (aged 24-65 years). We subdivided RA patients into those with (*n* = 17) and those without infections (*n* = 6) within a 2-year period. In order to reduce the effect of RA treatment, we only included patients receiving methotrexate monotherapy or no RA treatments at baseline. Soluble serum protein levels of B-cell/ASC factors were quantified by multiplex immunoassays.

**Results:**

We identified that (1) serum levels of soluble BCMA, APRIL, CD40, and CD40L were significantly decreased in RA patients relative to healthy individuals; (2) serum soluble BCMA, predominantly released by ASC, correlated with serum concentrations of class-switched immunoglobulins, IgG and IgA; and (3) RA patients with a history of infections had significantly lower soluble BCMA levels compared with healthy donors and with RA patients without infections.

**Conclusions:**

Our study using soluble factors linked to B-cell/ASC activation and survival suggests that there is a paucity of ASC in a subset of RA patients and that this may be linked to altered antibody production and increased risk of infections. Further delineating the link between ASC and infection susceptibility in RA may optimize disease management and provide novel insights into disease pathogenesis that are susceptible to intervention.

## 1. Introduction

Rheumatoid arthritis (RA) is the most common systemic autoimmune disease, affecting approximately 0.5-1% of adults worldwide [1]. It is characterized by synovial inflammation, autoantibody production, and systemic features [1, 2]. Although inflammatory manifestations predominate in RA, some affected individuals also have an intrinsically increased susceptibility to infections [2–4]. Indeed, a higher incidence of bronchitis and pneumonia, sometimes preceding joint disease [4, 5], higher frequency of tuberculosis (TB) [6], and increased mortality due to infections [3, 7] are described before steroids and immunomodulators were used. This is particularly concerning since RA patients usually require medications that further suppress pathways integral to immune function, and as a consequence, infections remain a leading complication in RA [8].

Although the association between RA and increased susceptibility to infections has long been recognized [4], the causes underlying this phenomenon remain poorly defined. Some patients with RA have hypogammaglobulinemia [9–13] suggesting that impaired production of antibodies could accompany the disease. Antibodies against infections are produced by antibody-secreting cells (ASC) including plasmablasts and plasma cells [14, 15]. However, autoimmunity can also result from anomalous production of autoantibodies by self-reactive ASC [2, 16, 17]. Therefore, ASC defects may play a dual role in the production of pathogenic autoantibodies as well as in deficient responses to infections in RA [14, 16, 17]. Additional support for this notion is provided by the study of primary antibody immunodeficiencies, in which RA and other autoantibody-associated diseases are more common [18–20].

To produce antibodies, B-cells must first mature into ASC, which may be short-lived effectors in early antibody responses or prolonged lifespan plasma cells that produce long-lasting, class-switched, highly specific antibodies (e.g., immunoglobulins A, G) [14, 15, 21]. Short-lived ASC are produced upon direct antigen stimulation or during early T-cell-dependent responses [14]. Long-lived ASC are generated in a complex process triggered by cell-to-cell interactions with follicular T-helper cells via CD40 and CD40 ligands (CD40L), followed by extensive proliferation of activated B-cells to generate germinal centers [14, 22, 23]. During this process, class-switch recombination (CSR) in B-cells occurs to produce antibody isotypes (e.g., IgA, IgG, and IgE) [14, 15, 22]. Germinal center reactions under T-cells help also cause the proliferation and selection of activated B-cells to produce highly specific antibodies against encountered antigens [14, 15]. After these processes, long-lived ASC producing antibodies of high affinity and variable isotypes are generated. During ASC generation, B-cells and ASC migrate in processes controlled by B-cell trafficking molecules such as CXCL9, CXCL10, and CXCL11 that, among other functions, home cells to inflamed tissues or CXCL13 that directs B-cell/ASC in germinal centers [14, 21]. Most newly differentiated B-cells undergo apoptosis unless survival signals are delivered by two members of the TNF receptor superfamily, BAFF (B-cell-activating factor) and APRIL (a proliferation-inducing ligand) via binding to their receptors TACI (transmembrane activator and calcium modulator and cyclophilin ligand interactor) [14, 24] and BAFF-R (BAFF receptor) [24, 25]. Another BAFF/APRIL receptor, BCMA (B-cell maturation antigen), is predominantly expressed by ASC and is essential to ensuring the prolonged lifespan of plasma cells [26, 27].

Although humoral immune responses have been extensively studied in RA, studies often focus on the role of ASC in the production of autoantibodies [1, 2, 28, 29]. It remains unclear whether ASC abnormalities in RA also alter normal protective antibody responses against infections or immunizations. The study of human ASC in rheumatologic diseases is limited by the fact that most, especially long-lived, plasma cells reside in the bone marrow and are usually not accessible in peripheral blood [14, 15, 21]. Thus, our knowledge about the mechanisms regulating the development and function of ASC in individuals with RA is incomplete.

The goal of this study was to study whether individuals with RA exhibit abnormalities in serum levels of soluble factors linked to critical steps for ASC generation, including those for B-cell activation and B-cell/ASC survival and trafficking. Specifically, we aimed to characterize differences in such factors between patients with RA and healthy individuals. We also sought to examine associations between some of those factors and clinical outcomes linked to ASC function, such as antibody production and infections.

## 2. Methods

### 2.1. Subjects

We conducted a retrospective evaluation of clinical data from patients included in the Johns Hopkins Arthritis Cohort (JHAC) [30]. The JHAC has detailed longitudinal clinical information in over 730 active RA patients followed during clinical visits and a biorepository containing serum samples from approximately 80% of enrolled subjects. For the present study, our patient population was drawn from individual RA subjects enrolled in the JHAC since 2012 (*n* = 277) with serum samples collected on the same day as a clinical assessment. We included all patients aged 18 to 65 years old, with a provisional RA diagnosis (physician assessment and use of disease-modifying antirheumatic drugs (DMARD)) or meeting American College of Rheumatology 2010 criteria for RA [31], receiving monotherapy with methotrexate (MTX) or taking no immunomodulators at serum sample collection. Serum samples were part of the JHAC biorepository. Demographic information and stored serum from 10 healthy control volunteers were also used. All samples and data were collected after informed written consent approved by the Johns Hopkins Institutional Review Board.

### 2.2. Clinical Data

Basic demographic and clinical characteristics (sex, age of onset, autoantibody status, disease duration, disease activity, medication use, and MTX dose) at the time of the baseline visit (when serum sample was collected) were extracted from the JHAC clinical database. Seropositive arthritis was defined as RA associated with positive rheumatoid factor (RF) or anticitrullinated protein antibodies (ACPA) as previously described. RA activity was determined using the Clinical Disease Activity Index (CDAI), classified as remission (CDAI ≤ 2.8), low disease activity (CDAI > 2.8 and ≤10), moderate disease activity (CDAI > 10 and ≤22), and high disease activity (CDAI > 22) as previously defined [32]. Disease onset was defined as the age at which RA symptoms started, and disease duration was established as the time since the onset of RA symptoms to the date of sample collection in years. Infectious events were self-reported using a patient questionnaire inquiring about joint/bursa, cellulitis/skin, sinusitis, diverticulitis, sepsis, pneumonia, bronchitis, gastroenteritis, meningitis/encephalitis, upper respiratory infections (URI), an urinary tract infection (UTI) at every visit. Infections not included in these categories were self-reported under “other infection.” Information on whether hospitalization and/or parenteral antibiotics associated with these infectious events was also recorded.

### 2.3. Measurement of B-Cell/ASC-Soluble Serum Factors and Immunoglobulins

Measurements of soluble factors involved in B-cell/ASC activation, survival, and homing as well as serum immunoglobulin concentrations were performed on serum samples from the JHAC biorepository. We quantified serum protein levels of soluble factors involved in B-cell activation (CD40, CD40L) and B-cell/ASC survival (APRIL, BAFF, sTACI, and sBCMA) or homing (CXCL10, CXCL11, and CXCL13) using a magnetic bead-based multiplex immunoassay (R&D Human Magnetic Luminex Assay). All samples, including healthy control samples, were processed simultaneously. Serum concentrations of immunoglobulins A (IgA), G (IgG), and M (IgM) in RA patients were assessed by nephelometry at the Johns Hopkins Hospital Clinical Immunology laboratory.

### 2.4. Statistical Analyses

Data were analyzed using the software STATA version 14 (StataCorp. *Stata Statistical Software: Release 14*. College Station, TX. 2015), R studio version 1.10.153 (RStudio Team (2018). RStudio: Integrated Development for R. RStudio Inc., Boston, MA), and Minitab Student version 14 (Minitab 14 Statistical Software (2018). Minitab Inc., State College, PA). Basic clinical characteristics of all RA patients were extracted from the JHAC clinical database. Age, age of onset, disease duration, disease activity (Clinical Disease Activity Index score (CDAI) score), and MTX dose are summarized as medians and interquartile ranges (IQR) as distributions were non-Gaussian and were compared using the Wilcoxon rank-sum test. Differences in sex, ethnicity, and autoantibody status between RA subgroups were contrasted using Fisher's exact test. Differences in B-cell/ASC activation, survival, and homing factors between RA patients and healthy controls (HC) were examined using a Wilcoxon rank-sum test, and Spearman's correlations were used to evaluate the relationship between sBCMA, serum immunoglobulins (IgA, IgG, and IgM) and selected B-cell/ASC activation, and survival factors (APRIL, CD40, and CD40L). Differences between HC and the two RA subgroups (RA with and without infections) were compared using nonparametric methods (Kruskal-Wallis tests to assess differences among groups and Wilcoxon rank-sum tests for pair-wise comparisons) or a chi-square test as indicated. Finally, since controls and RA patients were not age-matched, we built linear regression models to evaluate whether differences in B-cell/ASC activation, homing, and survival factors (using log-transformed values) between healthy controls and RA patients persisted after adjusting by age at baseline.

## 3. Results

### 3.1. Study Population

**Figure 1:**
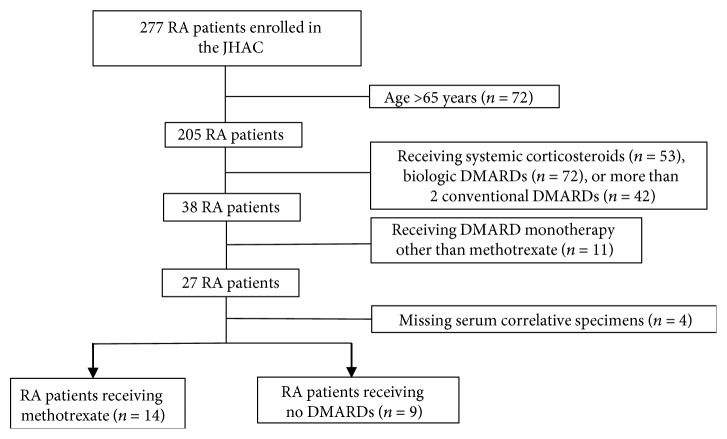
Flow diagram shows selection of patients included in our study. A total of 23 RA patients from the JHAC were included in the final analysis. Abbreviations: RA = rheumatoid arthritis; JHAC = Johns Hopkins Arthritis Cohort; DMARDs = disease-modifying antirheumatic drugs.

Clinical data from 277 patients enrolled in the JHAC database and biorepository were screened for eligibility. There were 205 subjects at serum sample collection who met age eligibility criteria (Figure 1). We excluded 53 patients receiving systemic corticosteroids, 72 receiving biologic DMARDs, and 42 treated with more than one conventional DMARD. We also removed patients taking sulfasalazine, as it can be associated with hypogammaglobulinemia or monotherapy with a conventional DMARD other than methotrexate (MTX). After excluding 4 eligible subjects with insufficient serum specimens, we included 23 RA patients in the final analysis (Figure 1).

The main demographic and clinical characteristics of subjects included are displayed in Table 1. The median age of RA patients was 55 years (IQR 16, range 24-64 years), with 19 females (83%) and 4 males (17%). Patients in our sample were predominantly Caucasian (*n* = 16, 70%). The median age of disease onset was 39 years (IQR 26), and median disease duration was 7 years (IQR 11). In regards to disease activity, patients had a median CDAI of 3.9 (IQR 9.5). Nine patients were in remission, six had low disease activity, and six had moderate (*n* = 4) or high disease activity (*n* = 2). There were two patients with no disease activity scores available (*n* = 2). We identified 14 patients treated with MTX only (61%) and 9 patients receiving no DMARDs (39%) at the time of serum sample collection. Healthy donors for this study included 10 healthy volunteers (*n* = 10) of both sexes aged 21 to 62 years (median 41 years, IQR 23) without history of significant medical conditions. No differences in age were detected among the three groups (*p* = 0.09); however, RA patients as a group were older when compared with healthy subjects (*p* = 0.03).

### 3.2. B-Cell Activation and Survival Factors Were Significantly Decreased in RA Patients Compared with Healthy Individuals

**Figure 2:**
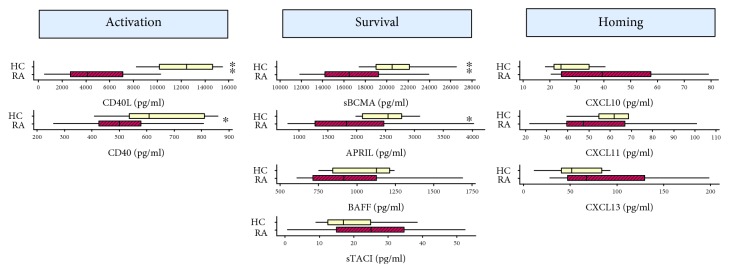
Box plots comparing serum B-cell factor protein levels in RA patients and healthy controls. Levels of each protein are indicated for healthy controls (HC, top yellow box; *n* = 10) and RA patients (RA, bottom red box; *n* = 23). The median value is indicated with a vertical line, with the box comprising the IQR, and whiskers representing the maximum and minimum values within the fences (±1.5 IQR). ^∗∗^ *p* < 0.01 and ^∗^ *p* < 0.05. Serum levels of B-cell activation factors (CD40, CD40L) and B-cell/ASC survival elements (sBCMA, APRIL) were significantly decreased in RA patients compared with healthy controls.

In order to examine differences in soluble factors released during ASC generation in RA patients, we evaluated serum levels of soluble factors involved in B-cell/ASC activation, survival, and homing in RA subjects (*n* = 23) and healthy individuals (*n* = 10). We identified significantly reduced levels of APRIL (median levels RA = 1,807.8 pg/ml vs. controls = 2,544.9 pg/ml, *p* = 0.009) and sBCMA (median levels RA = 16,535.2 pg/ml vs. controls = 20,487.8 pg/ml, *p* = 0.008), the ligand-receptor duo responsible for maintaining ASC survival, in the serum RA subjects relative to controls. We also found reduced serum levels of soluble CD40 (median levels RA = 482.3 pg/ml vs. controls = 608.3 pg/ml, *p* = 0.02) and CD40L (median levels RA = 4,446.3 pg/ml vs. controls = 12,450.5 pg/ml, *p* < 0.0001). Differences in sBCMA, CD40 and CD40L, and APRIL persisted after adjusting by age (Figure S1). Serum levels of soluble BAFF were slightly decreased in RA patients but were nonsignificantly different from healthy subjects in a bivariate analysis (median levels RA = 913.9 pg/ml vs. controls = 1128.0 pg/ml, *p* = 0.21) or after adjusting by age (*p* = 0.52) (Figure S1). In contrast with other survival factors, the soluble receptor TACI was increased in RA. Differences with healthy donors failed to achieve statistical significance in an unadjusted analysis (median levels RA = 26.56 pg/ml vs. controls = 16.9 pg/ml, *p* = 0.13) and after adjusting by age (*p* = 0.42) (Figure S1). There were nonsignificant increases in CXCL13 (median levels RA = 68.34 pg/ml vs. controls = 51.89 pg/ml, *p* = 0.24), in CXCL10 (median levels RA = 34.73 pg/ml vs. controls = 24.09 pg/ml, *p* = 0.07), and in CXCL11 (median levels RA = 47.30 pg/ml vs. controls = 61.90 pg/ml, *p* = 0.07) in the unadjusted comparison (Figure 2). However, the small increases seen in CXCL10 and CXCL13 in RA patients became statistically significant when adjusting by age (Figure S1).

### 3.3. Serum Soluble BCMA Protein Concentrations Had a Positive Correlation with Serum Levels of Class-Switched Immunoglobulins in RA Patients

**Figure 3:**
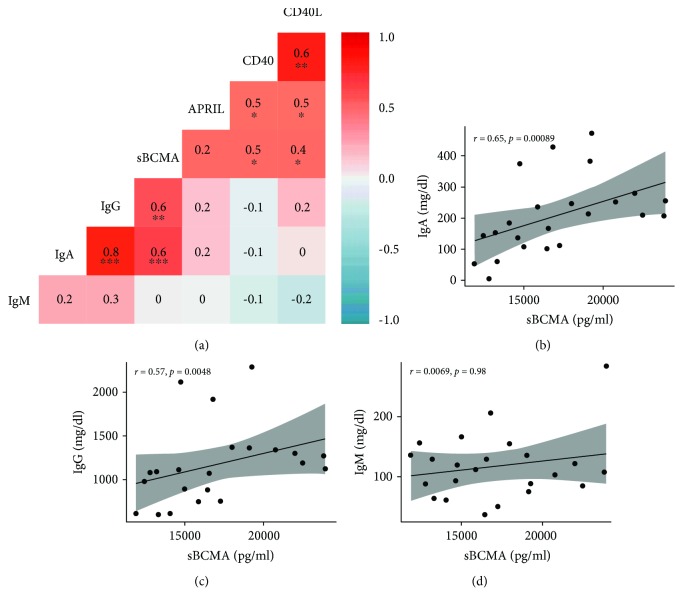
Correlation of B-cell factors and immunoglobulins in RA patients. (a) Spearman correlation matrix of soluble B-cell factors and Ig levels in RA. (b-d) Scatter plots demonstrating individual correlations between sBCMA and IgA (b), IgG (c), and IgM (d). Serum sBCMA (but not CD40, CD40L, or APRIL) had a positive correlation with serum IgG (rho = 0.6, *p* < 0.01) and IgA (rho = 0.6, *p* < 0.001), both ASC-derived class-switched antibodies, but not with IgM levels which is produced without class-switching. Moderate correlations were also seen between B-cell activation factors (CD40, CD40L) and sBCMA levels. ^∗^ *p* < 0.05, ^∗∗^ *p* < 0.01, and^∗∗∗^ *p* < 0.001.

Because of CD40/CD40L interactions, APRIL and BCMA mediate critical steps in ASC generation and survival; we examined correlations between serum levels of the above factors and of class-switched immunoglobulins, IgG and IgA, as well as IgM. Median IgA, IgG, and IgM for RA patients were 207 mg/dl (range 4.9-472 mg/dl), 1110 mg/dl (range 599-2290 mg/dl), and 112 mg/dl (range 37-284 mg/dl), respectively. Only serum sBCMA showed significant correlations with serum concentrations of class-switched immunoglobulins IgG (Spearman's rho = 0.6, *p* < 0.01) (Figure 3(b)) and IgA (Spearman's rho = 0.6, *p* < 0.001) (Figure 3(c)). There was also a strong correlation between serum IgA and IgG levels (rho = 0.8, *p* < 0.001). Conversely, there were no significant correlations between sBCMA and IgM (Spearman's rho = 0.0, *p* = 0.9 (Figure 3(d)) which is produced before class-switching. Finally, to a lesser degree, moderate correlations were found between sBCMA, CD40, and CD40L and between APRIL, CD40, and CD40L (Figure 3). Of note, there were three RA patients with mild IgG hypogammaglobulinemia (IgG < 640 mg/dl) [33] and one subject with undetectable IgA serum levels (IgA < 5 mg/dl). These four subjects had sBCMA measurements in the lowest quartile for RA patients and below the minimum serum sBCMA level found in normal subjects. There were also two subjects with elevated IgG and IgA levels defined as serum concentrations above 1349 mg/dl [33] and 312 mg/dl [33], respectively, in spite of having sBCMA levels below the lowest level found in healthy donors (17,420 pg/ml). These two patients were characterized by having seropositive disease with high titers of ACPA antibodies (>200 IU).

### 3.4. Patients with RA and Infections Had Lower Serum sBCMA Levels Compared with RA Subjects without Infectious Events and Healthy Subjects

**Figure 4:**
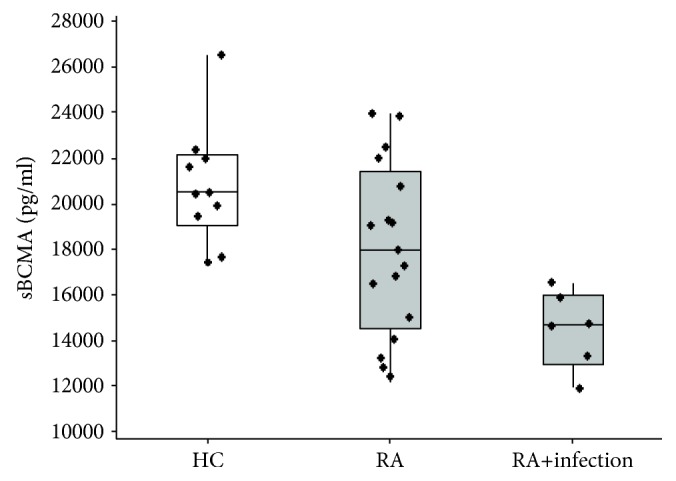
Box plots comparing serum sBCMA protein levels among RA patients with and without infections and healthy donors. Levels of sBCMA in healthy controls (HC, left white box; *n* = 10), RA patients without infections (middle gray box; *n* = 17), and RA patients with infections (right gray box; *n* = 6) are shown. Patients with RA with a history of infections had significantly lower sBCMA levels compared to RA subjects without a history of infections (*p* < 0.05) and to healthy controls (*p* < 0.01). ^∗∗^ *p* < 0.01 and^∗^ *p* < 0.05.

To investigate the clinical relevance of decreased serum sBCMA and its correlation with serum IgA and IgG levels, we examined the relationship between sBCMA and infections among RA patients. We subdivided the RA group into patients with a history of infections (*n* = 6) and those without infections (*n* = 17) within 2 years after collection of baseline serum samples. There were 10 infectious events reported during the follow-up period in six patients: 4 sinopulmonary infections, 2 influenza infections, 3 soft-tissue/mucosal infections, and 1 herpes zoster infection. We compared median serum sBCMA levels among the three groups (RA patients with infections (*n* = 6), RA patients without infections (*n* = 17), and healthy individuals (*n* = 10)). The median serum sBCMA level was 14,669.7 pg/ml (IQR 2553 pg/ml) in the RA subgroup with infections, 17,979.8 pg/ml (IQR 5777.3 pg/ml) in the uninfected RA subgroup, and 20,487.8 (IQR 2561.3 pg/ml) in healthy individuals, with statistically significant difference (*p* = 0.004) among these subgroups. Pair-wise comparisons revealed that RA patients with infections had significantly lower serum sBCMA levels compared with RA patients without infections (*p* < 0.05) and with healthy controls (*p* < 0.01) (Figure 4). The levels of serum sBCMA in uninfected RA patients compared with healthy controls were not significantly different (*p* = 0.063). To examine whether this pattern persisted in the absence of methotrexate, we conducted a similar analysis in untreated patients (Figure S2) and continued to observe significantly decreased sBCMA levels in RA patients with infections (*n* = 2) compared with healthy controls (*n* = 10) (*p* = 0.03). Serum sBCMA levels also were lower in patients with infections than in those without infections (*n* = 7), although this difference was not significant (*p* = 0.07).

### 3.5. Serum Immunoglobulins Were Not Associated with Infection Events in RA Patients

**Figure 5:**
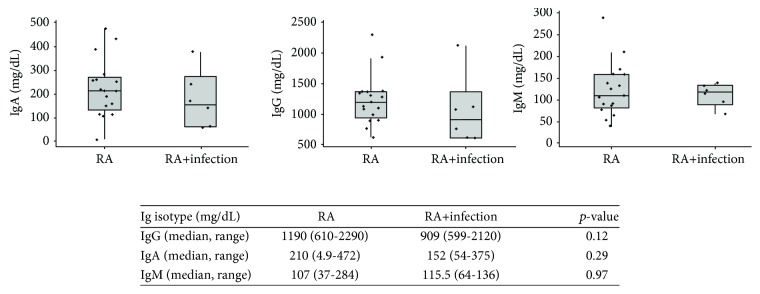
Box plots and analyses comparing serum immunoglobulins (IgG, IgA, or IgM) in RA patients with infections and without infections. The median value is indicated with a horizontal line, with the box comprising the IQR and whiskers representing the maximum and minimum values within the fences (±1.5 IQR). Comparisons between groups were performed using Wilcoxon rank-sum tests. No statistically significant differences were found in serum IgG, IgA, or IgM between the two patient groups.

As serum immunoglobulin is clinically used to evaluate the production of antibodies [33, 34], we evaluated whether serum levels of IgG, IgA, and IgM predicted infectious events in RA patients. We compared serum concentrations of IgG, IgA, and IgM between the two patient groups (RA patients with infections (*n* = 6), RA patients without infections (*n* = 17)) and evaluated the number of infections recorded within the subsequent 2 years after the initial immunoglobulin measurements. Patients with infections had median IgG, IgA, and IgM levels of 909 mg/dL (IQR 501), 152 mg/dl (IQR 177), and 115.5 mg/dl (IQR 36), respectively. Median serum IgG, IgA, and IgM were 1190 mg/dl (IQR 390), 210 mg/dl (IQR 111), and 107 mg/dl (IQR 71) in the group of RA patients without infections. As shown on Figure 5, there were a few subjects with IgG levels below normal adult ranges (<639 mg/dl) [33] in both groups and one subject with undetectable IgA levels in the group of RA patients without infections. IgM levels were all within normal adult range concentrations. Importantly, when median levels of the three immunoglobulin isotypes (IgG, IgA, and IgM) were compared between the two RA groups, no statistically significant differences were found (Figure 5).

## 4. Discussion

Rheumatoid arthritis is a disorder of immune dysregulation characterized by enhanced inflammation and increased susceptibility to infections [1, 2]. Autoantibody production suggests ASC dysfunction and is central to RA disease pathogenesis [1, 2, 35]. However, how ASC are dysregulated in RA remains inadequately defined. Our study is aimed at studying this phenomenon by examining soluble receptors and immune factors involved in critical steps of ASC generation and survival as a means to evaluate ASC activity in RA.

As most membrane-bound receptors and ligands of the TNF-receptor superfamily (e.g., CD40, CD40L, BAFF, APRIL, TACI, and BCMA) are cleaved from cell surfaces upon binding by their ligands, serum levels of these soluble factors are often interpreted as markers of cell activation [25, 27]. Most studies investigating factors involved in B-cell/ASC activation and survival in RA describe that they are often upregulated [25, 36–40]. In keeping with these observations, we identified several patients with soluble levels of the above markers exceeding the measurements seen in healthy subjects in our study. However, a group of RA patients had significantly decreased serum measurements of CD40, CD40L, BCMA, and APRIL, but not of BAFF, TACI, or B-cell homing factors in comparison with healthy controls. Considering the biological function of these elements, these findings suggested that key steps in mature B-cell activation and/or ASC survival could be impaired in a subset of RA patients.

To determine the clinical significance of the above findings, we next sought to examine the relationship of the above markers of B-cell activation and ASC survival with immunoglobulin production and occurrence of infections, two clinical outcomes related to ASC function [14]. Interestingly, we found that serum sBCMA, but not CD40, CD40L, or APRIL, correlated with serum levels of IgG and IgA, class-switched antibodies, usually produced by long-lived plasma cells [14], but not with serum IgM, which is produced before class-switching, and it is the major antibody of short-lived, primary antibody responses [14]. In turn, decreased serum sBCMA levels were also associated with increased frequency of infections in RA patients. Indeed, all RA patients with an infection in our dataset had serum sBCMA levels below the lowest level observed in healthy subjects and were significantly decreased compared to RA subjects without infectious events. In spite of the small sample, we continued to observe this trend among untreated RA patients showing that low serum sBCMA levels occur even in the absence of medications. Taken together, since sBCMA is released predominantly by ASC, and in its membrane-bound form, it is the main survival receptor on the surface of long-lived plasma cells [15, 26, 27]; these findings suggested that a paucity in ASC activity and possibly in ASC survival could underlie deficient production of protective antibodies and increased susceptibility to infections in the subgroup of RA patients characterized by lower serum sBCMA.

Defects in ASC function in RA may result from primary disease pathogenesis, secondary to the disease course or to treatments or as a combination of the above. For instance, if low serum sBCMA levels reflect paucity of BCMA at the cell level, ASC survival could be compromised [26]. Alternatively, BCMA could be the result of impaired ASC generation and hence a consequence of decreased cell counts [27]. Specifically, as suggested by the observed decrease in soluble CD40L levels, anomalous CD40L-mediated costimulation by T-helper cells could impair ASC formation and production of IgG and IgA [23, 41]. Intrinsic B-cell defects at antigen recognition, signaling through the B-cell receptor, or at B-cell costimulatory checkpoints could result in deficient B-cell activation and ASC generation [14, 21, 42]. The demonstration of decreased sBCMA serum levels in patients with primary antibody deficiencies and inherited defects in these pathways lend support to this notion [43]. Later in the process, failure to complete ASC differentiation can result from dysregulation of transcription factors that preserve B-cell identity (e.g., PAX5, PU.1-IRF-8, and BACH2) [14, 44] or that promote B-cell transformation into ASC (e.g., BLIMP1, XBP1, or IRF4) [14, 44]. Previous descriptions of RA risk loci on genes involved in the regulation of ASC generation (e.g., IRF4, IRF8, and BLIMP1) [45, 46] support a role for these factors in RA. On the other hand, changes occurring in the natural history of RA or in response to RA treatment could affect ASC activity and impact serum sBCMA release. For instance, cell exhaustion as a consequence of chronic inflammatory disease may blunt B-cell activation and ASC activity [47, 48]. Likewise, methotrexate, as a folate antagonist, may contribute through impairment of B-cell proliferation [49]. In summary, our findings that serum sBCMA is differentially expressed in a subset of patients with RA prompt multiple considerations of how these changes occur and underscores the need for additional investigation into the mechanisms governing ASC generation and survival as well as secondary events affecting ASC function in RA.

We note that serum immunoglobulin concentrations, including those of IgG and IgA, were not associated with the occurrence of infectious events in patients with RA. A possible explanation for this observation is that autoantibodies represent a constitutive fraction of serum immunoglobulins [28, 50] in RA; thus, total IgG and IgA levels may rise if high levels of autoantibodies are present [51]. This idea is supported by our finding that patients with high serum IgG and IgA levels in the setting of low sBCMA had high titers of ACPA antibodies. This observation raises the question as to whether serum immunoglobulin concentrations may not accurately reflect the production of protective antibodies against infections in patients with circulating autoantibodies and additional functional testing of humoral immune responses (e.g., measurements of immunizations responses) and/or additional measurements of ASC activity such as sBCMA should be used to evaluate humoral immunity against infections in RA.

Our study has several limitations. The main caveat of our approach is that the detection of soluble mediators in serum does not necessarily imply that they are functional [25], and additional studies at the cellular level are required to establish the origins of the observed changes. As a cross-sectional study, temporal and causal relationships cannot be established, and our retrospective analysis of cohort data may also be at risk of selection, recall, and attrition bias. Additionally, because of the small sample, our analyses did not include additional factors that modify immune responses in RA such as disease activity or medication use. Nonetheless, we describe significant differences in biologically relevant mediators of B-cell activation (CD40 and CD40L) and ASC survival (sBCMA) and suggest that these findings may be common in RA. Moreover, we have demonstrated an association between a marker of ASC activity, serum sBCMA, and relevant outcomes that are biologically and clinically associated with ASC function such as production of class-switched immunoglobulins and occurrence of infections. These findings prompt further investigation into ASC biology in RA, as these clinically relevant outcomes may relate to anomalies at the cellular level that are susceptible to intervention [25].

## 5. Conclusion

Our study suggests that deficient ASC generation or survival occurs in a subset of RA patients, a novel finding. Elucidating how ASC are regulated in RA may offer novel insights into disease pathogenesis. We establish if soluble immune receptors and factors linked to ASC activity may be used to predict ASC-related clinical events (e.g., infection risk, vaccination responses, and autoantibody production) and inform personalized strategies to modify those outcomes. Furthermore, examining the interplay between ASC function and immunomodulatory drugs may guide a better choice of immunosuppressants and, in the future, provide the basis upon which new preventive and therapeutic approaches are developed.

## Figures and Tables

**Table 1:** Basic demographic and clinical characteristics of RA patients and RA subgroups.

	Healthy donors *N* = 10	RA patients without infections *N* = 17	RA patients with infections *N* = 6	*p* value
*Gender*				
Male/female	1/9	4/13	0/6	0.54
*Ethnicity*, *n* (%)				
White/Caucasian	6	13 (76.5)	3 (50)	
Non-white	4	4 (23.5)	3 (50)	0.33
*Age in years*				
Median (IQR)	41 (26)	54 (15)	55.5 (9)	0.09
*Disease duration in years*				
Median (IQR)		5 (6)	10.5 (11)	0.32
*Age of onset*				
Median (IQR)		39 (25)	41.5 (20)	0.97
*Autoantibody status*				
Seropositive/seronegative RA		14/3	4/2	0.57
*Disease activity (CDAI score)*				
Median (IQR) [range]		2.95 (10.2) [0-37]	8.5 (4.0) [0-22]	0.34
Number of patients		16	5	
*RA medication category*				
MTX-treated/no DMARD		10/7	4/2	1.0
*Methotrexate dose (mg/week)*				
Median (IQR)		17.5 (10)	22.5 (2.5)	0.30
Number of patients		9	3	

Abbreviations: RA = rheumatoid arthritis; IQR = interquartile range; CDAI score = Clinical Disease Activity Index score.

## Data Availability

The datasets generated and/or analyzed during the current study are available from the corresponding authors on reasonable request.

## Supplementary Materials

Supplementary Materials We are submitting two additional figures as supplementary material to this manuscript. Supplementary Figure 1 displays scatter plots with individual data points of B-cell/ASC soluble markers by age in RA patients and controls. Supplementary Figure 2 displays the comparison of sBCMA levels among controls and untreated RA patients with and without infections.
