# Supplementary material for: Soluble Markers of Antibody Secreting Cell Function as Predictors of Infection Risk in Rheumatoid Arthritis
Source: J Immunol Res. 2019 Apr 28;2019:3658215. doi: 10.1155/2019/3658215 (PMC6512050; doi:10.1155/2019/3658215)
Supplement: Supplementary Materials — We are submitting two additional figures as supplementary material to this manuscript. Supplementary Figure 1 displays scatter plots with individual data points of B-cell/ASC soluble markers by age in RA patients and controls. Supplementary Figure 2 displays the comparison of sBCMA levels among controls and untreated RA patients with and without infections. [file 3658215.f1.pdf]

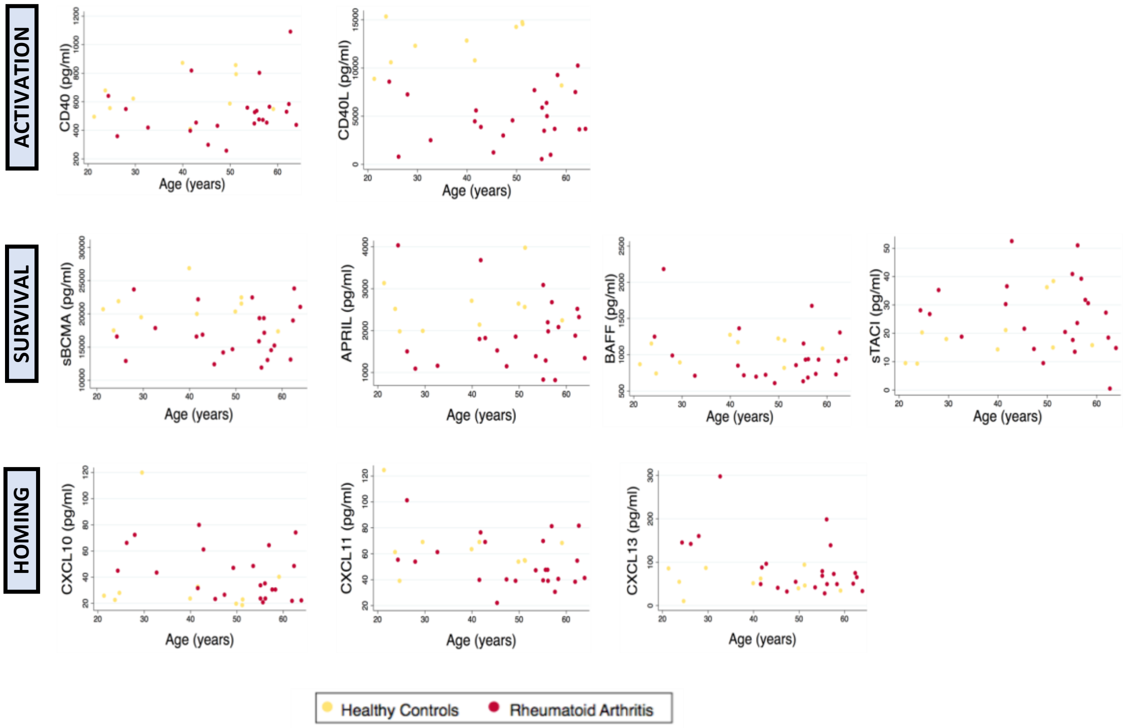


| | B-cell/ASC soluble factors (log-transformed scale) | | | | | | | | |
|---|---|---|---|---|---|---|---|---|---|
| | CD40 | CD40L | sBCMA | APRIL | BAFF | sTACI | CXCL10 | CXCL11 | CXCL13 |
| Unadjusted | **0.057** | **0.000** | **0.007** | **0.015** | 0.357 | 0.523 | 0.162 | 0.072 | 0.118 |
| Adjusted | **0.028** | **0.000** | **0.011** | **0.024** | 0.517 | 0.422 | **0.046** | 0.227 | **0.027** |

**Figure S1.** B-cell/ASC activation, survival and homing soluble factors between patients with RA (n=23) and healthy controls (n=10)**.** P-values of unadjusted and adjusted comparisons are displayed. There were statistically significant increased levels of CD40, CD40L, sBCMA and APRIL in RA patients that persisted after analyses were adjusted by age. Mild increases in CXCL10 CXCL13 in the RA group became statistically significant in the adjusted analysis.
* Unadjusted analyses performed using non-parametric Wilcoxon rank-sum tests using raw soluble factor values in pg/ml

** Analyses performed using multiple linear regression with log-transformed values of B-cell/ASC activation, survival and homing soluble factors and adjusted by age

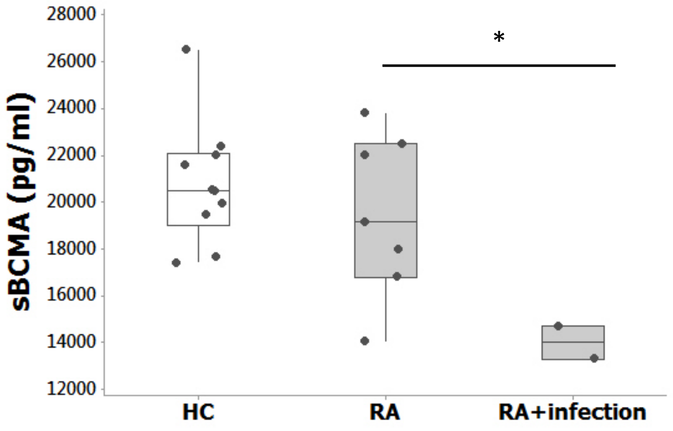


**Figure S2. Box plots comparing serum sBCMA protein levels among untreated RA patients with and without infections and healthy donors.** Levels of sBCMA in healthy controls (HC, left white box; n=10), untreated RA patients without infections (middle gray box; n=7), and untreated RA patients with infections (right gray box; n=2) are shown. Patients with RA with a history of infections had significantly lower sBCMA levels compared to healthy controls (p<0.03). When comparing the two arthritis subgroups (RA with and without infections), we had very few patients and the difference between median serum sBCMA was not significant (p=0.07), however, serum sBCMA levels were lower in untreated RA patients with infections following the trend previously seen between these two groups (Fig. 4). *p<0.05.
